# Association Between Physical Activity and Breakfast Skipping Among Saudi Adolescents: A Multi-City Analysis of the 2019 Global School-Based Student Health Survey

**DOI:** 10.3390/children13091198

**Published:** 2026-09-04

**Authors:** Abdulrahman I. Alaqil, Mohummed H. Alkhraiji

**Affiliations:** 1 Department of Physical Education, College of Education, King Faisal University, Al-Ahsa 31982, Saudi Arabia; 2 Department of Exercise Physiology, College of Sport Sciences and Physical Activity, King Saud University, Riyadh 145111, Saudi Arabia; moalkhraiji@ksu.edu.sa

**Keywords:** physical activity, breakfast skipping, adolescents, Saudi Arabia, Global School-based Student Health Survey

## Abstract

**Background**: Physical activity and breakfast consumption are commonly clustered adolescent health behaviors, but Saudi evidence on their association is limited to a single city, and whether this relationship varies by sex or age remains untested. **Objective**: We aimed to examine the association between physical activity frequency and breakfast skipping among Saudi adolescents and determine whether it differs by sex and age. **Methods**: This cross-sectional study used 2019 Global School-based Student Health Survey (GSHS) data from adolescents in five Saudi cities (Riyadh, Jeddah, Dammam, Khamis Mushait, Tabuk; N = 5900). Cases with missing data on any analysis variable were excluded listwise (final N = 5431). Physical activity frequency and breakfast skipping were both assessed using self-reported GSHS questionnaire items. Logistic regression via generalized estimating equations (clustered on school) estimated adjusted odds ratios, adjusting for sex, age (years), grade, nationality, and city; moderation by sex and age was tested with interaction terms and a joint Wald test, and robustness was assessed with three sensitivity analyses. **Results**: The final analytic sample (N = 5431) had a mean age of 13.71 years (standard deviation (SD) 1.13) and was 54.6% female. Breakfast-skipping prevalence was 18.7%. Each additional physically active day was associated with 10% lower odds of breakfast skipping (adjusted odds ratio [AOR] = 0.90, 95% confidence interval [CI] 0.86–0.93, *p* < 0.001), a finding robust across all sensitivity analyses, including multiple imputation (OR = 0.89, 95% CI 0.87–0.92). Female sex, non-Saudi nationality, older age, and city of residence (higher odds in Jeddah and Dammam relative to Riyadh) were independently associated with greater odds of skipping. Neither sex nor age moderated the physical activity–breakfast association (joint Wald χ^2^(2) = 2.70, *p* = 0.259). **Conclusions**: Physical activity frequency was independently and robustly associated with lower breakfast skipping among Saudi adolescents, irrespective of sex or age, extending single-city evidence to a larger and more diverse population. School-based strategies that jointly promote physical activity and breakfast consumption may not require tailoring by sex or age, although this study did not evaluate intervention effectiveness, and this inference should be confirmed prospectively; city-level variation suggests monitoring and resource allocation may benefit from geographic disaggregation.

## 1. Introduction

A central premise of adolescent health research, dating to Jessor’s psychosocial framework of problem behavior, is that risk behaviors rarely occur in isolation: they co-occur because they are organized by a common underlying disposition toward conventional or unconventional living, rather than arising from independent, unrelated causes [1]. Three decades later, large-scale surveillance data confirmed this empirically on a global scale. Analyzing 304,779 adolescents across 89 countries, Uddin and colleagues found that 82.4% reported two or more of six major non-communicable disease risk behaviors simultaneously, with physical inactivity clustering most strongly alongside low fruit and vegetable intake in both sexes [2]. The more useful scientific question, then, is which specific behaviors cluster, for whom, and why.

Physical activity and breakfast consumption are two behaviors frequently implicated in this clustering pattern, and each carries substantial independent health weight. Fewer than one in five adolescents worldwide meet the World Health Organization’s recommendation of 60 min of daily moderate-to-vigorous physical activity, a shortfall that has changed little in two decades and is consistently more pronounced among girls than boys [3,4], a sex gap also documented among adults in the same region and period [4]. Breakfast omission, meanwhile, has been linked to elevated total and abdominal adiposity independent of sleep duration in multi-country adolescent cohorts, and represents a measurable loss of daily micronutrient and energy intake at a life stage of rapid growth [5]. Because both behaviors are anchored to the same morning window, one governed by household routine, parental oversight, and the adolescent’s own capacity for self-regulation, clustering theory would predict that adolescents who reliably engage in one are more likely to reliably engage in the other, positioning the physical activity–breakfast relationship as a specific, testable instance of the broader co-occurrence phenomenon described above [1,2].

Empirical tests of this specific pairing, however, have not consistently confirmed the prediction. A nationally representative National Health and Nutrition Examination Survey (NHANES) analysis of US adolescents found no association between breakfast skipping and meeting physical activity guidelines once confounders were adjusted for [6], and a Croatian cohort reported that physical activity did not mediate the relationship between breakfast consumption and adiposity in either sex [7]. In contrast, a more recent adolescent sample found a modest but significant association between physical activity and breakfast skipping whose strength differed by sex [8]. Applying the same Global School-based Student Health Survey (GSHS) instrument used in the present study, a Vietnamese national analysis similarly found that physical inactivity clustered with other lifestyle risk behaviors in sex-specific patterns, reinforcing that clustering structure, and not merely prevalence, can itself vary by population and by sex [9]. Collectively, this body of work suggests that whether, and how strongly, physical activity and breakfast skipping travel together is not a fixed biological fact but a population-specific empirical question, one that Western evidence alone cannot resolve for other cultural and schooling contexts.

Saudi Arabia is a population in which this question carries direct public health weight, since both behaviors are independently prevalent there. A synthesis of 18 Saudi studies found physical activity prevalence ranging from 4% to 44.5%, with breakfast skipping and low fruit and vegetable intake among the most commonly reported unhealthy habits [10], and subsequent surveillance confirmed meaningful variation in these behaviors by age, sex, and region within the Kingdom [11]. Using the Arab Teens Lifestyle Study 2 in Riyadh, Al-Hazzaa and colleagues reported that 53% of adolescents were physically inactive and 65.7% did not eat breakfast daily, with non-daily breakfast intake significantly associated with lower physical activity in multivariable analysis [12,13], direct evidence that, in this single city at least, the two behaviors do cluster together as clustering theory would predict. Comparably high breakfast-skipping rates in Saudi primary-school children in Jeddah (approximately 80%) [14] and in Saudi-national young men in Riyadh (86.5%, the highest of any nationality studied) [15] indicate that this pattern was not confined to a narrow age band. The cultural determinants that plausibly shape breakfast consumption in this setting include close parental oversight of morning routines, sex-segregated school schedules that structure the timing of meals and commuting, and family meal practices tied to broader religious and cultural observance, including altered household eating schedules during Ramadan, which fall outside the reference period captured by this survey but may shape year-round family routines around meals [16].

What remains unclear is whether the association previously observed in Riyadh represents a broader feature of adolescent behavior in Saudi Arabia or reflects characteristics specific to a single urban setting, including its school environment, climate, and socioeconomic context. It is also unknown whether the strength of this association varies by sex or age, particularly given evidence that health-risk behaviors may cluster differently across demographic subgroups [2,9]. To our knowledge, no previous study has examined the association between physical activity frequency and breakfast skipping across multiple Saudi cities using a standardized Global School-based Student Health Survey framework while formally evaluating sex- and age-based effect modification and accounting for the clustered school-based sampling structure [17,18].

The present study addresses this gap using 2019 GSHS data collected from adolescents across five major Saudi cities: Riyadh, Jeddah, Dammam, Khamis Mushait, and Tabuk. Specifically, this study aims to (1) examine the association between physical activity frequency and breakfast skipping among Saudi adolescents; (2) determine whether this association differs by sex and by age. In doing so, this study extends a single-city finding into a substantially larger and more geographically diverse sample, and, by testing effect modification directly, provides the first evidence on whether a uniform or a sex/age-tailored approach is warranted for school-based interventions in this population that jointly target physical activity and breakfast consumption.

Sex and age were selected a priori as moderators of primary interest because both are established correlates of adolescent physical activity and dietary behavior [3], with the dietary component specifically supported by a systematic review of sex differences in adolescent eating motivations [19], while GSHS-based studies further indicate that patterns of behavioral co-occurrence may differ by sex rather than simply differ in prevalence [2]. This raises the possibility that the association between physical activity and breakfast skipping may not be uniform across demographic subgroups. Accordingly, we hypothesized that higher physical activity frequency would be associated with lower odds of breakfast skipping (H1) and that this association would be moderated by sex, with a stronger inverse association among males than females [8] (H2). We additionally examined age as an exploratory moderator without specifying a directional hypothesis, given the limited evidence regarding age-specific effect modification.

## 2. Materials and Methods

### 2.1. Study Design and Setting

A cross-sectional study was conducted between October and December 2019 across five cities representing five administrative regions of Saudi Arabia: Riyadh (Central region), Jeddah (Western region), Dammam (Eastern region), Khamis Mushait (Southern region) and Tabuk (Northern region). These cities were purposively selected to capture variation in socioeconomic status, urbanization, and climate across the Kingdom. A stratified multi-stage sampling procedure was used within each city. Each city was divided into four geographic areas (north, south, east, and west), and within each area, two public intermediate schools, one male-only and one female-only, consistent with Saudi Arabia’s sex-segregated schooling system, were randomly selected from the Ministry of Education school database using an online random-number generator. This yielded a total of 40 schools (20 male-only, 20 female-only; 8 schools per city). Eligible participants were students enrolled in the selected public intermediate schools; enrollment was not restricted by nationality, and both Saudi and non-Saudi students attending the sampled schools were eligible to participate. Sample size calculated using G*Power 3.1.9.2 (power = 80%, α = 0.05), with the effect size derived from the average difference in adherence to moder-ate-to-vigorous physical activity (MVPA) recommendations (21% in males, 13% in females; Cohen’s w = 0.106) reported across four prior Global School-based Student Health Survey (GSHS) studies conducted in Gulf Cooperation Council countries; this calculation indicated a minimum requirement of 699 participants under simple random sampling; to account for the clustered, school-based sampling frame, a design effect of 5.0 (intraclass correlation of approximately 0.027 at the mean cluster size of 147.5 students per school) was applied, yielding a minimum requirement of approximately 3462 participants, rounded up to 3500 for planning purposes, across the five cities, a target the achieved sample substantially exceeded. Ethical approval was obtained from the College of Medicine Institutional Review Board (IRB) at King Saud University (E-19-3976). All procedures adhered to the principles of the Declaration of Helsinki. In addition, approval was given by the Ministry of Education in each city. Written informed consent was obtained from the parents or legal guardians of all students involved in the study. Student assent was also obtained prior to participation. Student assent was also obtained prior to participation, consistent with the distinct ethical functions of parental consent and child assent in pediatric research [20]. Approval to conduct the study was obtained from the participating schools before data collection.

#### 2.1.1. Data Collection

Questionnaires were distributed to the selected schools in each city by two trained physical education supervisors (one male, one female). Participants were instructed to complete the questionnaire independently at home, without input from parents, siblings, or friends, and were informed that responses were anonymous and would not be shared with school staff. Completed questionnaires were returned to a nominated teacher the following day; participants who missed this deadline but still wished to participate were permitted to return their questionnaire within the same week. Completed questionnaires were collected from each school by the assigned supervisor and forwarded to the principal research team.

#### 2.1.2. Instrument

Data were collected using a self-administered questionnaire adapted from the WHO Global School-based Student Health Survey (GSHS) [18], modified from the version validated in Bahrain (2016) and translated into Arabic for administration in this population [21], following standard guidelines for the cross-cultural adaptation of self-reported instruments [22]. The questionnaire covered sociodemographic characteristics, anthropometry, dietary behavior, personal hygiene, road and personal safety practices, psychosocial wellbeing, physical activity, sedentary behavior, and sleep. The present analysis draws specifically on the physical activity and breakfast-consumption items described below; the remaining modules were not analyzed for the purposes of this study.

#### 2.1.3. Physical Activity

Physical activity was assessed with the standard GSHS item: “During the past 7 days, on how many days were you physically active for a total of at least 60 min per day?” Participants were provided with a definition of MVPA as any activity that increases heart rate and causes breathlessness some of the time, with examples including running, fast walking, swimming, cycling, playing soccer, and aerobics [23]. Response options ranged from 1 (0 days) to 8 (7 days); for analysis, responses were recoded to reflect the actual number of active days (0–7).

#### 2.1.4. Breakfast Intake

Breakfast consumption was assessed with a single item asking how often, in the past 30 days, participants had eaten breakfast, with five response options: never, rarely, sometimes, most days, and always. For analysis, breakfast skipping was defined a priori as a binary outcome, coded 1 for participants reporting breakfast consumption “never” or “rarely” and 0 for those reporting “sometimes,” “most days,” or “always.” This threshold follows the classification used in prior breakfast-skipping research in comparable adolescent samples [6,7].

#### 2.1.5. Statistical Analysis

This cross-sectional study was conducted and is reported in accordance with the STROBE (Strengthening the Reporting of Observational Studies in Epidemiology) guideline for observational studies. Analyses were performed using R software version 4.4.1 (R Foundation for Statistical Computing, Vienna, Austria) and RStudio (2026.07.1+147) (Posit PBC, Boston, MA, USA). Continuous variables are presented as mean ± standard deviation (SD), and categorical variables as frequency and percentage (%) with 95% confidence intervals (95% CI). Analyses used the complete dataset (N = 5900); no respondents were excluded a priori, and cases missing data on any analysis variable were excluded listwise. Physical activity was operationalized as days per week (0–7) with ≥60 min of activity; breakfast skipping was defined a priori as a binary outcome (never/rarely vs. sometimes/most days/always) from the 5-category, 30-day item described above, with 6 out-of-range responses (0.1%) recoded as missing. The primary complete-case analysis assumes data are missing completely at random; the multiple-imputation sensitivity analysis relaxes this to the less restrictive assumption of data missing at random conditional on the variables included in the imputation model; neither assumption can be formally verified, but the close correspondence between the complete-case and multiply-imputed estimates for the primary association was reassuring with respect to the plausibility of the missing-at-random assumption. Since students were sampled within schools, the primary model was a logistic regression estimated via generalized estimating equations (GEE; exchangeable working correlation, robust sandwich standard errors, clustered on school), yielding population-averaged adjusted odds ratios (AORs). Model 1 regressed breakfast skipping on physical activity, adjusting for sex, age (years, centered), grade, nationality, and city (Riyadh as reference). Because age and grade are structurally related (Pearson r = 0.755 in this sample), variance inflation factors (VIFs) were computed for the full Model 1 design matrix to assess multicollinearity; all VIFs were below 3 (age, VIF = 2.47; Grade 2, VIF = 1.63; Grade 3, VIF = 2.88; all other covariates < 1.6), below the conventional threshold of concern (VIF > 5), and both variables were retained as they capture conceptually distinct developmental and school-structural constructs. Model 2 added physical activity × sex and physical activity × age terms to test moderation, evaluated via a joint 2-df Wald χ^2^ test as the pre-specified confirmatory test, with sex- and age-group-stratified models reported descriptively. Three sensitivity analyses assessed robustness: a non-clustered logistic regression; a proportional-odds model on the full 5-category breakfast outcome; and a multiple-imputation-by-chained-equations re-estimation (20 imputations, predictive mean matching, Rubin’s rules) using the full sample. Generalized estimating equations were fitted using the geepack package (2026.07.1+147) [24]; the proportional-odds sensitivity model was fitted using the MASS package [25]; and multiple imputations were performed using the mice package [26]. Statistical significance was defined as a two-tailed α of 0.05, with no multiplicity correction applied given the single pre-specified primary and moderation hypotheses.

## 3. Results

The final analytic sample (N = 5431) ranged in age from 11 to 18 years (mean 13.71, SD 1.13) and was 54.6% female (Table 1). Of 5900 adolescents, 469 (7.9%) had missing data on one or more analysis variables and were excluded listwise (final N = 5431; participant flow shown in Supplementary Figure S1). Excluded respondents were more often male, slightly older, and less physically active than those retained (all *p* < 0.05); a multiple-imputation sensitivity analysis nonetheless produced a virtually identical estimate for the primary association (OR = 0.89, 95% CI 0.87–0.92) to the complete-case result, indicating no material bias from missingness. Breakfast-skipping prevalence was 18.7% (Table 1). Skippers were disproportionately female, non-Saudi, and less physically active than non-skippers (all *p* < 0.001), and skipping prevalence varied significantly by city (*p* < 0.001); groups did not differ by age or grade.

**Figure 1:**
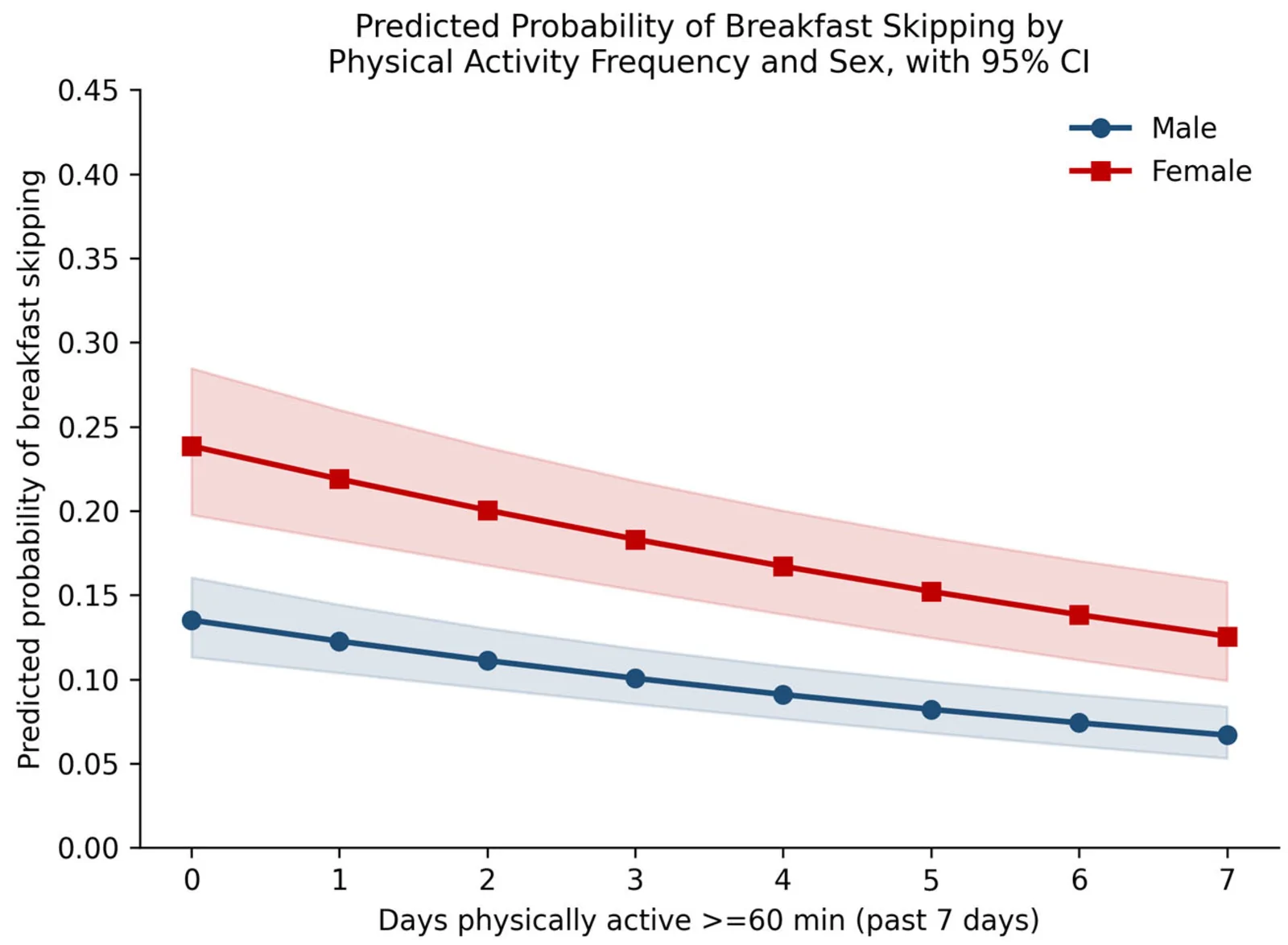
Model-predicted probability of breakfast skipping by physical activity frequency and sex (Model 1), with 95% confidence bands (shaded), holding grade, nationality, and city at their reference/mean values.

Each additional physically active day was associated with 10% lower odds of breakfast skipping (AOR = 0.90, 95% CI 0.86–0.93, *p* < 0.001; Table 2, Model 1), independent of sex, age, nationality, and city; predicted probabilities across the full range of physical activity frequency, by sex, are shown in Figure 1. Female sex, non-Saudi nationality, residence in Jeddah or Dammam (vs. Riyadh), and older age were independently associated with higher skipping odds; a Benjamini–Hochberg correction applied across the ten Model 1 coefficients confirmed that age’s association remained significant at the corrected threshold (raw *p* = 0.021, corrected threshold = 0.025). This association was robust across all sensitivity analyses (non-clustered logistic regression OR = 0.90; ordinal model OR = 1.11 per day; both *p* < 0.001). Neither sex nor age moderated this association: the interaction terms were consistent with no effect (physical activity × sex: AOR = 1.00, 95% CI 0.93–1.07; physical activity × age: AOR = 1.02, 95% CI 1.00–1.05). This was confirmed by a non-significant joint Wald test (χ^2^(2) = 2.70, *p* = 0.259; Table 2, Model 2). Sex-stratified (male AOR = 0.90; female AOR = 0.89) and age-stratified estimates were directionally consistent, with attenuation observed only in the small oldest subgroup (16–18 years, n = 246, *p* = 0.530).

## 4. Discussion

This study established three principal findings. First, physical activity frequency was inversely and independently associated with breakfast skipping among Saudi adolescents. Second, this association was not moderated by sex or by age, indicating a consistency across these subgroups that had not previously been formally tested in this population. Third, breakfast-skipping prevalence varied significantly by city, independent of individual characteristics, an unanticipated finding once the geographic variable was correctly resolved. All three findings were drawn from a substantially larger and more geographically diverse sample than previously available for this question, and the first finding in particular proved robust across three sensitivity analyses targeting distinct threats to validity: school-level clustering, dichotomization of the outcome, and the pattern of missing data.

The principal finding is consistent with problem behavior theory [1] and with global evidence that physical inactivity and poor dietary behavior co-occur in adolescent populations rather than arising independently [2,9]. It also does more than replicate the only direct prior Saudi evidence on this pairing. Al-Hazzaa and colleagues reported a significant physical activity–breakfast association among adolescents in Riyadh [12,13]; the present study shows that this relationship extends across a substantially larger and more geographically diverse sample, supplying the external validity that a single-city finding cannot establish on its own.

This result also diverges instructively from the Western literature, where a nationally representative NHANES analysis of US adolescents found no such association [6] and a Croatian cohort found that physical activity did not mediate the breakfast–adiposity relationship [7]. A plausible, if unconfirmed, explanation is contextual. Saudi adolescents’ mornings are typically structured by comparatively close parental oversight, gender-segregated schooling, and family meal practices with strong cultural standing [16]. These conditions may bind physical activity and breakfast consumption to the same underlying behavioral regularity more tightly than the looser, more autonomous routines common among Western adolescents, among whom greater self-directed food choice has itself been linked to less regular breakfast consumption [27]. This account is consistent both with the mechanism problem behavior theory proposes [1] and with a systematic review identifying parental breakfast consumption and two-parent family structure as the family correlates most strongly and consistently associated with adolescent breakfast consumption across settings [28]. It nonetheless remains an inference from context rather than a tested pathway in the present data, and should be treated as a hypothesis for future work rather than an explanation already demonstrated.

The absence of moderation by sex or age was itself a substantive finding, not merely a null result to be set aside. It stands against a recent multi-country study reporting sex-specific differences in this same association [8] and against GSHS-based clustering evidence showing sex-differentiated patterns of behavioral co-occurrence elsewhere [9]. One reading was that, in this population, the upstream determinants shared by physical activity and breakfast consumption, household routine and school structure, operate similarly for boys and girls and across the age range studied here, rather than being channeled through sex- or age-specific pathways. A more conservative reading was that the study was better powered to detect a main effect than a moderation effect of realistic size, particularly in the oldest stratum (16–18 years), which comprised a small fraction of the sample; the attenuated estimate observed there was as consistent with reduced precision as with a genuine absence of effect, and this age range merits dedicated, adequately powered follow-up before either reading is accepted. A minimum-detectable-effect-size calculation favors the former reading: at the achieved sample of 5431, the two-degree-of-freedom joint test had 80% power at α = 0.05 to detect an effect as small as Cohen’s w = 0.04, well below the interaction estimates actually observed, supporting the absence of moderation as a genuine finding for the sample as a whole rather than a failure of power. The same calculation restricted to the oldest stratum alone (16–18 years, n = 246) raises the minimum detectable effect approximately 4.7-fold, to a Cohen’s w near 0.18, sufficient on its own to account for the attenuated estimate observed in that subgroup without invoking a genuine age-specific reversal, consistent with general guidance that effect-size and interval estimates, not *p*-values alone, should anchor the interpretation of null or borderline findings [29].

The city-level variation, higher skipping odds in Jeddah and Dammam than in Riyadh, net of sex, age, grade, and nationality, was not a pre-specified hypothesis and was reported with corresponding caution. Differences in urban food environments, commuting distance and school start times, or regional socioeconomic composition not captured by the available covariates were all plausible contributors. Dammam and Jeddah are more industrialized and commercially developed than Khamis Mushait and Tabuk, which could plausibly translate into denser fast-food and convenience-food environments, longer parental commuting times that reduce structured family breakfast time and later or more variable school start times. These city-level structural differences were not measured in the present survey and are offered as hypotheses rather than established explanations. A recent study of adolescents in three of the five cities in the present study found that perceived neighborhood safety independently predicted physical activity levels, with female adolescents reporting safety-related barriers significantly more often than males, suggesting that city-level differences in the built environment could plausibly extend to the behaviors examined here as well [30,31]. It was also possible that the differential rates of missing data observed across cities play some part, although the multiple-imputation sensitivity analysis indicated that missingness did not materially bias the primary physical-activity estimate overall. Adjudicating among these explanations lies beyond the scope of the present analysis, but linking this survey to city-level environmental or socioeconomic indicators was a natural next step.

This study’s principal strengths lie in its scale and its methodological transparency. It draws on a substantially larger and more geographically diverse Saudi adolescent sample than has previously been available for this question, using a standardized instrument with documented reliability in this population [17]. The clustering-aware analytic strategy was verified empirically rather than assumed, and the central finding held across three independent sensitivity analyses targeting different sources of potential bias, a degree of triangulation that lends the result more weight than any single model could provide on its own.

Several limitations temper these conclusions. The cross-sectional design cannot establish temporal order: regular physical activity may foster more structured eating routines, regular breakfast consumption may supply the energy and routine that sustains activity, or both may follow from an unmeasured third factor such as parental structure or household socioeconomic status. Additionally, sampling was restricted to public intermediate schools; adolescents attending private schools, who may differ systematically in socioeconomic status and associated lifestyle behaviors, were not represented, and the findings should not be extrapolated to this subgroup. Although participants were instructed to complete the questionnaire independently, compliance with this instruction in the home setting could not be verified, and the presence of parents or siblings during completion may have introduced social-desirability bias in either direction for both the physical activity and breakfast items. Both variables were self-reported, and self-reported physical activity and dietary behavior in adolescents are known to be susceptible to recall error and social desirability bias [32], a source of measurement error this design cannot rule out; objective measures such as accelerometry or interviewer-administered dietary recall would strengthen future replications [33]. Relatedly, several lifestyle variables available elsewhere in the GSHS instrument, including sedentary behavior, sleep duration, and body mass index, were not included as covariates, reflecting the study’s a priori scope and, for body mass index specifically, the high missingness and data-quality concerns affecting the underlying anthropometric items; residual confounding by these unmeasured factors cannot be excluded and should be addressed in future analyses with more complete anthropometric data. A small number of response codes for two covariates, grade and nationality, extended beyond what the available questionnaire appendix could document and were retained under provisional labels; these should be confirmed against the master GSHS instrument before the findings are generalized further. Finally, five cities, however diverse, do not constitute a nationally representative sample, and extension of these findings to rural or unstudied regions of the Kingdom should not be assumed.

These findings bear directly on ongoing national policy. Saudi Arabia’s Vision 2030 Quality of Life Program has set an explicit target of increasing weekly physical activity participation among the population, and two of its instruments, the National Diet and Physical Activity Strategy (2015–2025) and the Twenty-Four-Hour Movement Practice Guidelines for Saudi Arabia, already treat physical activity and dietary behavior as a joint rather than separate target for children and adolescents [34]. The present results offer direct empirical support for that combined framing: in this population, physical activity and breakfast consumption move together, and because no evidence of moderation by sex or age emerged, school-level implementation of these existing strategies may not need to be differentiated by sex or age to reach both boys and girls across the adolescent years; we emphasize that this inference concerns the association observed here and not the effectiveness of any specific intervention, which was not evaluated in this study. Systematic evidence on school-based physical activity interventions cautions, however, that behavioral effects are typically modest and are strongest when programs are theory-informed, delivered by trained personnel, and involve parents [35], a design principle with particular relevance where delivery relies on the shared-routine mechanism proposed here, in which parental oversight and household morning structure jointly support both regular breakfast consumption and regular physical activity engagement. A national policy review has separately identified a lack of multisectoral coordination and inconsistent monitoring across Saudi physical activity initiatives [34]; the significant city-level variation documented in the present study suggests that disaggregating monitoring and evaluation by city, rather than treating the Kingdom as behaviorally uniform, may sharpen where these existing strategies are working and where they are not.

Taken together, these results support the premise with which this study began: that adolescent health behaviors are better understood as interconnected than as isolated. Physical activity and breakfast consumption travel together in this population much as problem behavior theory would predict, and they do so with a consistency across sex and age that narrows, rather than widens, the questions future research needs to ask. What remains open is not whether these behaviors are related in Saudi adolescents, but why, and whether policy that already treats them as a linked pair can be sharpened, through family- and school-based delivery and city-level monitoring, to move both together. Future research should also examine whether breakfast consumption mediates the relationship between physical activity and broader cardiometabolic outcomes in Saudi adolescents, extending the present behavior-to-behavior association toward downstream health endpoints.

## 5. Conclusions

Physical activity frequency was independently and robustly associated with lower odds of breakfast skipping among adolescents across five major Saudi cities, irrespective of sex or age, extending evidence previously limited to a single city to a larger, more diverse population. Since this association did not vary by sex or age, school-based programs that jointly promote physical activity and breakfast consumption, such as active-morning initiatives paired with school breakfast provision, may not require separate versions for boys and girls or for different grade levels to reach the intended population. The city-level variation observed instead suggests that monitoring and resource allocation for existing national strategies would benefit from disaggregation by city rather than treating the Kingdom as behaviorally uniform, allowing implementers to identify where, specifically, additional investment is needed. Policy implementation based on these cross-sectional findings should await confirmatory evidence from longitudinal and intervention studies, as detailed in the Discussion.

## Figures and Tables

**Table 1:** Sociodemographic characteristics and physical activity levels of adolescents in Saudi Arabia, overall and by breakfast-skipping status (N = 5431).

Characteristic	Overall (N = 5431)	Breakfast Skippers (n = 1014)	Non-Skippers (n = 4417)	*p*	Skipping Prevalence Within Group, %	SMD
Sex, n (%)						
Male	2463 (45.4)	311 (30.7)	2152 (48.7)	<0.001	12.6	h = 0.290
Female	2968 (54.6)	703 (69.3)	2265 (51.3)		23.7	
Age, years, mean (SD)	13.71 (1.13)	13.73 (1.11)	13.70 (1.14)	0.376	-	d = 0.031
Nationality, n (%)						
Saudi	4470 (82.3)	792 (78.1)	3678 (83.3)	<0.001	17.7	h = 0.134
Non-Saudi	961 (17.7)	222 (21.9)	739 (16.7)		23.1	
City, n (%)						
Riyadh	1129 (20.8)	171 (16.9)	958 (21.7)	<0.001	15.1	
Jeddah	1082 (19.9)	233 (23.0)	849 (19.2)		21.5	
Dammam	1350 (24.9)	306 (30.2)	1044 (23.6)		22.7	
Khamis Mushait	1188 (21.9)	179 (17.6)	1009 (22.8)		15.1	
Tabuk	682 (12.6)	125 (12.3)	557 (12.6)		18.3	
Grade, n (%)						
1st intermediate	2209 (40.7)	392 (38.7)	1817 (41.1)	0.344	17.7	
2nd intermediate	1564 (28.8)	304 (30.0)	1260 (28.5)		19.4	
3rd intermediate	1658 (30.5)	318 (31.4)	1340 (30.3)		19.2	
Physically active days (0–7/week), mean (SD)	2.35 (2.36)	1.86 (2.27)	2.46 (2.36)	<0.001	-	d = −0.257

SMD = standardized mean difference (Cohen’s d for continuous variables, Cohen’s h for binary variables) between breakfast skippers and non-skippers; for sex and nationality, the SMD was computed from the skipping-prevalence-within-group column in the same row (e.g., 12.6% in males vs. 23.7% in females), consistent with the conditioning direction used throughout this table.

**Table 2:** Adjusted odds ratios for the association between physical activity frequency and breakfast skipping among Saudi adolescents, including tests of effect modification by sex and age (generalized estimating equations models).

Predictor	Model 1 AOR (95% CI)	Model 2 AOR (95% CI)	*p* (Model 1)
Physical activity (per day/week, 0–7)	0.90 (0.86–0.93)	0.89 (0.85–0.94) ^†^	<0.001
Sex: Female vs. Male	2.01 (1.72–2.35)	2.02 (1.65–2.48) ^†^	<0.001
Age, years (centered)	1.12 (1.02–1.23)	1.07 (0.98–1.18) ^†^	0.021
Nationality: Saudi vs. non-Saudi	0.82 (0.71–0.95)	0.82 (0.71–0.95)	0.009
City: Jeddah vs. Riyadh	1.30 (1.04–1.64)	1.30 (1.04–1.64)	0.023
City: Dammam vs. Riyadh	1.45 (1.21–1.74)	1.45 (1.21–1.73)	<0.001
City: Khamis Mushait vs. Riyadh	0.97 (0.80–1.19)	0.97 (0.80–1.19)	0.798
City: Tabuk vs. Riyadh	1.15 (0.84–1.58)	1.15 (0.84–1.57)	0.371
Grade 2 vs. Grade 1	1.04 (0.87–1.25)	-	0.670
Grade 3 vs. Grade 1	0.96 (0.74–1.23)	-	0.738
PA days × Sex (Female)	-	1.00 (0.93–1.07)	0.970
PA days × Age	-	1.02 (1.00–1.05)	0.118

AOR = adjusted odds ratio; CI = confidence interval. ^†^ Model 2 estimates for physical activity, sex, and age are conditional (evaluated at the reference/mean level of the interacting term) and are not directly comparable in magnitude to Model 1′s marginal estimates.

## Data Availability

The data presented in this study are available on request from the corresponding author. The data are not publicly available due to privacy or ethical restrictions.

## Supplementary Materials

The following supporting information can be downloaded at: https://www.mdpi.com/article/10.3390/children13091198/s1, Figure S1: Participant flow.
