# Supplementary material for: Association Between Physical Activity and Breakfast Skipping Among Saudi Adolescents: A Multi-City Analysis of the 2019 Global School-Based Student Health Survey
Source: Children (Basel). 2026 Sep 4;13(9):1198. doi: 10.3390/children13091198 (PMC13605389; doi:10.3390/children13091198)

Participant Flow

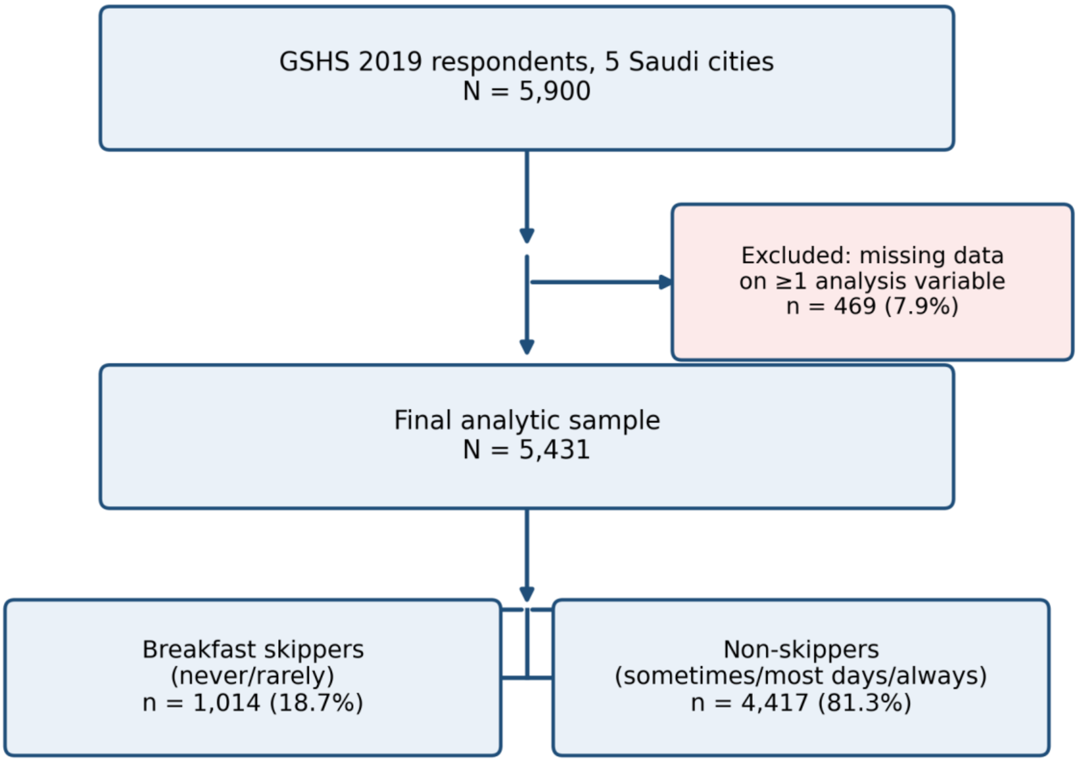

Supplement: Supplementary file 1 [file children-13-01198-s001.zip › children-4507262-supplementary.pdf]
